# Impact of Ruxolitinib Interactions on JAK2 JH1 Domain Dynamics

**DOI:** 10.3390/ijms26083727

**Published:** 2025-04-15

**Authors:** Hong Nhung Vu, Ragousandirane Radjasandirane, Julien Diharce, Alexandre G. de Brevern

**Affiliations:** 1 Université Paris Cité and Université de la Réunion, INSERM, EFS, BIGR U1134, DSIMB Bioinformatics Team, F-75015 Paris, France; hong-nhung.vu@etu.u-paris.fr (H.N.V.); ragousandirane.radjasandirane@etu.u-paris.fr (R.R.); julien.diharce@u-paris.fr (J.D.); 2 Université Paris Cité and Université de la Réunion, INSERM, EFS, BIGR U1134, DSIMB Bioinformatics Team, F-97715 Saint Denis Messag, France

**Keywords:** essential thrombocythemia, polycythemia vera, myeloproliferative neoplasm, splenomegaly, Janus kinase inhibitor, protein structure, protein ligand interaction, phosphorylation, molecular dynamics, structural alphabet, protein blocks

## Abstract

Janus kinase 2 (JAK2) is an important intracellular mediator of cytokine signaling. Mutations in the JAK2 gene are associated with myeloproliferative neoplasms (MPNs) such as polycythemia vera (PV) and essential thrombocythemia (ET), while aberrant JAK2 activity is also associated with a number of immune diseases. The acquired somatic mutation JAK2 V617F (95% of cases of PV and in 55–60% of cases of ET), which constitutively activates the JAK2, is the most common molecular event in MPN. The development of specific JAK2 inhibitors is therefore of considerable clinical importance. Ruxolitinib is a JAK inhibitor recently approved by the FDA/EMA and effective in relieving symptoms in patients with MPN. Ruxolitinib binds to the JAK2 last domain, namely JH1; its action on the dynamics of the domain is still only partially known. Using Molecular Dynamics simulations, we have analyzed the JH1 domain in four different states as follows: (i) alone, (ii) with one phosphorylation, (iii) adding Ruxolitinib, and (iv) with five phosphorylations and Ruxolitinib. The ligand induces a dynamic behavior similar to the inactive form of JH1, with a less flexible state than the phosphorylated active form of JH1. This study highlights the inhibitory effect of Ruxolitinib on the JH1 domain, demonstrating the importance of dynamics in regulating JH1 activation.

## 1. Introduction

Protein kinase is a very diverse, complex, and essential protein family. They play key roles in regulating cellular processes such as proliferation, survival, signaling, metabolism, and homeostasis [1,2,3]. However, they are also often deregulated by mutations that can lead to several kinds of diseases, especially cancers. As a result, they are in great demand as valuable drug targets. Janus kinase (JAK), often referred to as “just another kinase”, is a family of intracellular, non-receptor tyrosine kinases that transmit cytokine-driven signals through the JAK-signal transducers and activators of the transcription (JAK-STAT) pathway [4]. Janus kinase 2 (JAK2) phosphorylates the JAK-signal transducer and STAT proteins. These phosphorylations lead to the dimerization and activation of STATs through SH2-domain interactions [5]. STATs play a key role in processes like cell proliferation, differentiation, and immune responses, which are all essential for cell survival. Furthermore, the JAK/STAT pathway is recognized as one of the twelve major cancer pathways, highlighting the importance of proper JAK2 regulation in maintaining normal cell function. This suggests comprehending the fundamental processes of this pathway as thoroughly as possible [6,7].

JAK2 is a multi-domain protein composed of four consecutive domains as follows: FERM, SH2, the pseudokinase domain JH2, and kinase domain JH1 (see Figure S1). JAK2 undergoes trans-autophosphorylation in the activation loop of its kinase domain JH1, specifically on Tyr1007 and Tyr1008 residues [8,9]. While the exact purpose of this phosphorylation is not fully understood, it is believed to facilitate the recruitment and phosphorylation of STATs, thus, it is directly related to the activity of JAK2.

Constitutively active JAK2 has been observed in various cancer types, including breast cancer, lymphomas, and myeloid malignancies [10,11,12,13,14]. The V617F mutation [15], [16] is the most frequently detected mutation in myeloproliferative neoplasms (MPNs), found in over 97% of Polycythemia Vera (PV) patients [15] and more than 50% of cases of essential thrombocythemia [17] and primary Myelofibrosis (MF) [18,19,20].

A working diagnosis is considered when a JAK2 mutation is present alongside hemoglobin/hematocrit levels of >16.5 g/dL/49% in men or 16 g/dL/48% in women. Current treatments for PV have not been shown to extend the lifespan or prevent disease progression to post-PV MF or acute myeloid leukemia. Instead, current therapies focus on managing thrombosis and symptoms [21]. For instance, PV patients require periodic phlebotomy to maintain a hematocrit target of <45% and a daily dose of aspirin (81 mg). Cytoreductive therapy is reserved for high-risk cases, with first-line treatments including hydroxyurea and pegylated interferon-α, and second-line options being the use of Busulfan and Ruxolitinib [22,23].

Certainly, similar to many health conditions, certain patients may not respond to therapy or may develop resistance. Therefore, more specific agents are needed. In this context, Ruxolitinib, an oral inhibitor of Janus kinases 1 and 2, was first approved by the US Food and Drug Administration in 2011 for the treatment of intermediate- or high-risk myelofibrosis (MF), including primary MF, post-PV MF, and post-essential thrombocythemia MF. The approval was based on the positive efficacy and safety results from the COMFORT Phase 3 randomized controlled trials [24,25]. Under the trade names Jakavi or Jakafi (for the USA), it remains the standard medication for high-risk MF, with dose optimization and careful management playing a key role in maximizing its clinical benefits while ensuring patient safety. Interestingly, Ruxolitinib seems to have an important long-distance inhibitory effect on the JAK2 protein, since it does not act at the level of the mutated JH2 pseudokinase domain, but interacts with the JH1 kinase domain, by competing to the ATP-binding site for inhibiting the kinase function. Although the structure of the JAK2 JH1 domain in complex with Ruxolitinib is available, its impact at the atomic level has been underexplored. However, JAK2 is an important drug target and the understanding of the molecular mechanisms that govern the inhibition by Ruxolitinib could be crucial for new drug designs projects. The limited information we have about this system is the analysis of the interaction between JAK2 and Ruxolitinib, which takes place entirely in the binding cavity. The stabilization of the ligand is performed primarily by hydrophobic interactions, with 11 JAK2–ligand contacts involved [26].

Therefore, the aim of this work is to enhance our understanding of the impact of Ruxolitinib on the JH1 domain of JAK2. We will describe the difference between the dynamics of this domain with and without Ruxolitinib, as well as address the question of the effect of phosphorylation, using molecular dynamics simulation approaches.

## 2. Results

### 2.1. Overview of Systems

A total of 101 PDB entries correspond to the complete JH1 domain of the JAK2 kinase. Interestingly, all of them are in complex with small molecules such as Baricitinib, used in the treatment of rheumatoid arthritis and alopecia areata [27,28,29]; Fedratinib, used in the second wave for MPN [9,30,31]; or Lestaurtinib, which had been tested unsuccessfully for several cancers [32,33]. Two solved structures contained Ruxolitinib, namely PDB entries 6WTN [34] and 6VGL [29]. They are equivalent and both have good-quality resolution (1.9 Å). We selected the second one that was complete from residue 843 to 1131. A comparison against all the other available structures shows that there is low variability between the available data.

**Figure 1:**
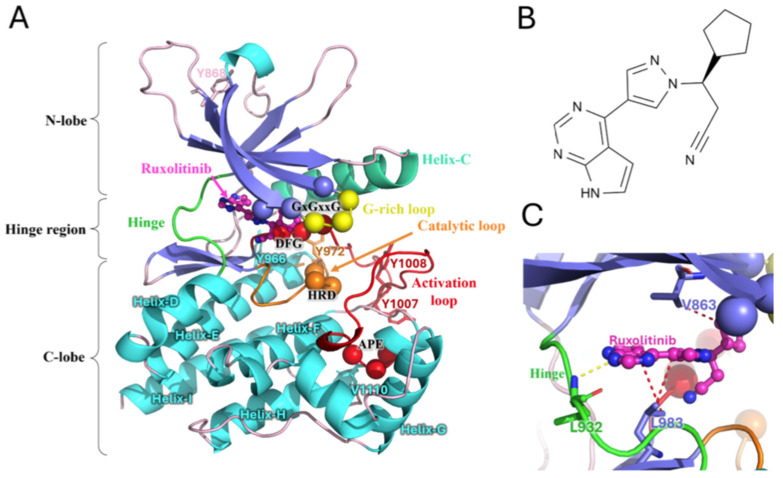
Visualization of the JH1 domain of JAK2 in complex with Ruxolitinib (PDB code: 6VGL). (**A**) JH1 subdomains: the activation loop (red), the catalytic loop (orange), the C-helix (teal blue), the G-rich loop (yellow), the hinge region (green), conserved motifs of JH1 (in spheres, the color depends on the region to which they belong), and the distribution of residues involved in the phosphorylation or interaction with Ruxolitinib (for the sticks, the color depends on the region in which they belong). Areas that do not belong to the indicated subdomains are colored according to the type of their secondary structure, as follows: ß-sheets (purple), α-helices annotated from D to I (cyan), loop (powder pink). (**B**) The chemical structure of Ruxolitinib. (**C**) The interaction of JH1 and the inhibitor Ruxolitinib identified by the PLIP (Protein-Ligand Interaction Profiler) [35]. Ruxolitinib is stabilized by hydrophobic interactions (red dashes) and hydrogen interactions (yellow dashes), the residues involved in these interactions are shown as sticks.

Figure 1 shows the different domains and sections of JH1 (see also Figure S2). As a kinase domain, it can be divided into three sections, namely the N-lobe, hinge region, and C-lobe (see Figure 1A). The C-lobe is mainly composed of helices (Helix-D, Helix-E, Helix-F, Helix-G, Helix-H, and Helix-I), an activation loop with two phosphorylation sites (Tyr1007 and Tyr1008), and the catalytic loop. One can notice that Tyr1007 is phosphorylated in this resolved structure (noted hereafter as pTyr1007). The N-lobe is known for the four ß-strands and Helix-C. The C- and N-lobes are connected by the so-called hinge region, which encompasses the ATP binding site, facilitating the protein’s function and being the area at which Ruxolitinib binds. The upper part of the binding cavity is composed of ß-strands (see Figure 1B,C [35]) and the well-known G-rich motif (GxGxxG). This glycine-rich loop in the ATP-binding site is one of the most highly conserved sequence motifs in protein kinases. The first and second glycines of this triad are invariant and essential to the protein’s function [36,37].

The UniProt entry of JAK2 (namely O60674) presents five potentially phosphorylated tyrosines (pTyrs), as follows: three are predicted by autocatalysis by similarity at positions 868, 966, and 972; and two at positions 1007 and 1008 have been experimentally demonstrated [38]. Those last mutations have been proven to be essential for the kinase functions of JAK2. Moreover, it had been shown that a number of lysine residues stabilize the conformation of pTyr 1007 (Lys 1005, Lys 1009, and Lys 1030) and pTyr 1008 (Lys 999) [38]. By consequence, four JH1 systems were considered in this study and were built from the selected structure as follows: (i) the apo form, with no phosphorylation and without Ruxolitinib; (ii) apo-pTyr1007, with the resolved pTyr1007, but without Ruxolitinib; (iii) Ruxo-pTyr1007 with pTyr1007 and in complex with Ruxolitinib (i.e., as in the PDB entry); and (iv) Ruxo-MultiPhosp is the domain with the five proposed pTyrs.

### 2.2. JAK2 JH1 Apo

**Figure 2:**
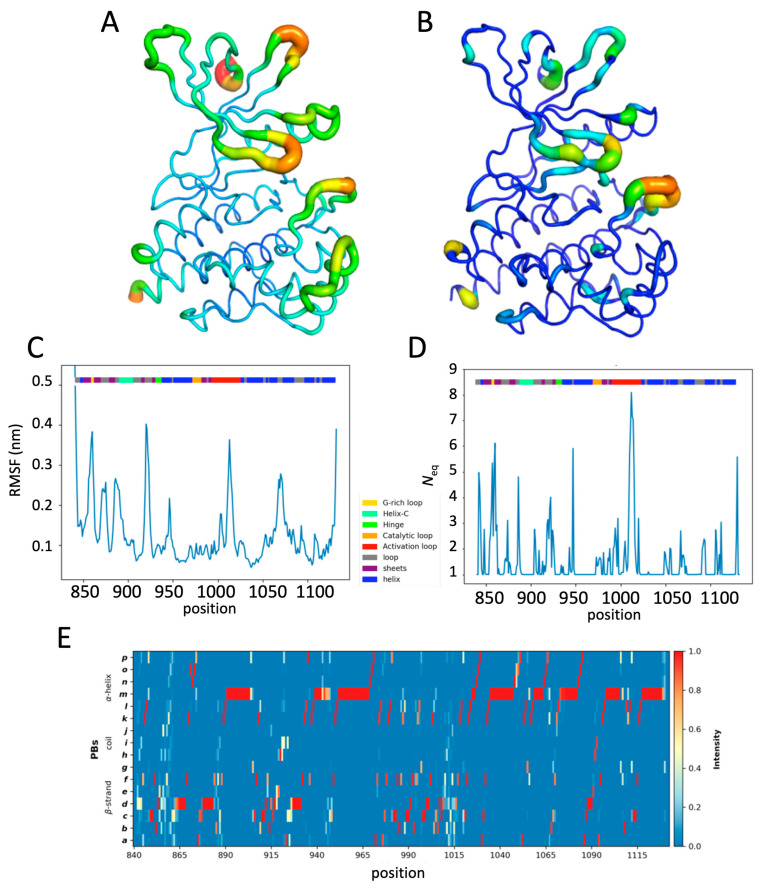
Analysis of the JH1 apo system. (**A**) RMSF values and (**B**) *N*_eq_ values of the apo system. Colors range from dark blue (minimum value) to red (maximum value). The size of the ribbon also depends on the values as follows: very thin means minimal and wide means high. (**C**) RMSF values and (**D**) *N*_eq_ values of the apo system along the residue position. A color gradient is put on top of the plots to define their positions (yellow: G-rich loop; cyan: helix C; green: hinge; orange: catalytic loop; red: activation loop; gray: loop; purple: ß-strands; and blue: other helices). (**E**) Protein block map of the apo system. The gradient on the right-hand side of the map goes from zero frequency (deep blue) to 100% (red).

Figure 2 shows the dynamics analysis of all molecular dynamics simulations of the apo JH1 system, using RMSF (see Figure 2A,C and Figure S3A), Protein Blocks (see Figure 2E and Figure S4), and *N*_eq_ (see Figure 2B,D and Figure S3A), arising from PBs. The system has a maximum RMSF at position 920, located in the loop between strands ß4 and ß5, for a fluctuation value about 4 Å. Two other flexible positions are located in the G-rich loop (positions 858–860) and in the activation loop (positions 1013–1014), with an RMSF greater than 3 Å. There is no position in the catalytic loop with an RMSF higher than 3 Å. The N-terminal part is mainly flexible because it is the beginning of the protein outside of most secondary structures.

Several differences arose from the RMSF and *N*_eq_ analysis, despite the fact that both are designed to estimate the dynamics of a position. However, it is important to note that RMSF is considered a global measure, while *N*_eq_ (based on PBs that are five residues long) is a rather local one. The correlations between the two are always quite low; here, it is only 0.24 (see Figure S3B).

Protein blocks [39] are interesting because they allow us to analyze the dynamics by categorizing the residues from total rigidity (*N*_eq_ = 1.0) to very flexible regions (*N*_eq_ > 6) or even disordered regions (*N*_eq_ > 8) [40]. The apo form presents a little more than half of the residues as being rigid (i.e., 53.2% with a *N*_eq_ of 1.0), 28.7% as quite rigid (*N*_eq_ between 1.0 and 2.0), 13.0% with a *N*_eq_ between 2.0 and 4.0, 3.4% as flexible with a *N*_eq_ between 4.0 and 6.0, 1.4% as extremely flexible with a *N*_eq_ between 6.0 and 8.0 and one position (1012) with a *N*_eq_ of 8.09, which is therefore considered as disorder. The regions are, therefore, very flexible to disordered, located in the N-terminus (842–843) and C-terminus (around 1130) domains, ß-strand and G-rich loop (857–860), different connecting loops (886, 922, and 947), and the activation loop (1010–1015), in particular. This proves that using these two measures (RMSF and *N*_eq_) gives distinct and complementary information (see Figure S3A). These are in agreement for the activation loop, which has the highest RMSF and a *N*_eq_ associated with a disordered region. There is also an agreement for a good flexibility towards ß-strands ß4 and ß5 with a very high RMSF and *N*_eq_ more than 6. However, for the helix region that has a high RMSF, the *N*_eq_ value is weak, showing that the local deformations, which impact the calculation of the RMSF, are just a question of weak rearrangement.

### 2.3. Impact of the Phosphorylation of pTyr1007 on JH1 Dynamics

**Figure 3:**
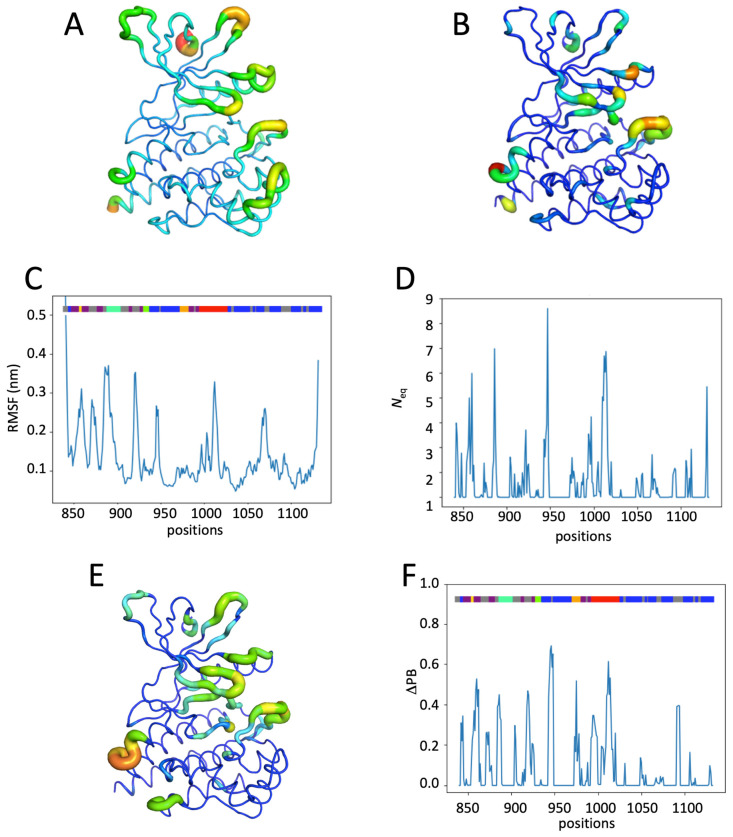
Analysis of the JH1 apo-pTyr1007 system. (**A**) RMSF values and (**B**) *N*_eq_ values of the apo-pTyr1007 system. (**C**) RMSF values and (**D**) *N*_eq_ values of the apo-pTyr1007 system along the residue position. ΔPB between the apo and apo-pTyr1007 system (**E**) on the JH1 domain and (**F**) along the sequence (**E**) on the JH1 domain and (**F**) along the sequence (see the Figure 2 legend for more details).

The system with the JH1 domain with the phosphorylated residue 1007 has a flexibility that is very similar to the system without phosphorylation (see Figure 3A–D and Figure S5A). Only two regions have differences in terms of fluctuation, as follows: (i) positions 885–890 of the C-helix, which have a RMSF greater than 3.5 Å, whereas the RMSF at the same positions in the apo system was only about 2.5 Å (see Figure 2A,C and Figure 3A,C). Position 890 thus has a RMSF of 3.7 Å, and (ii) region 920 (loop) has slight rigidification with a loss of 1 Å of the fluctuation value. At the N-terminal (positions 858–859), there is also a slight rigidification. The general *N*_eq_ analysis gives a distribution in terms of category quite close to what was seen for the apo system (52.9% rigid, 1.4% very flexible, 0.4% disordered; see Figure 2B,D and Figure 3B,D). However, a more precise analysis (see Figure 3B,D and Figure S5B) shows that the most important value is now position 950 (*N*_eq_ of 8.70), and that of position 1007 (the one that is phosphorylated) decreases slightly while remaining extremely flexible (*N*_eq_ value of 7). The C-helix area also shows a significant increase towards the very high flexibility category. The remaining regions are very similar to the non-phosphorylated apo system.

The ΔPB [41] allows us to go further in the comparison of the systems by analyzing the difference in PB distribution at each position between two systems. Thus, a ΔPB of zero means that for a given position, the observed PBs have exactly the same distribution, whether it is for a *N*_eq_ of 1 (same PB seen 100% of the time for the first and the second system) or 16. The ΔPB value goes up to 2 in the case that the observed PB (s) is totally different. Here, the ΔPB values between the apo- and mono-phosphorylated systems reach 0.60 (one-third of the PBs are distinct between the two systems) for positions having a strong *N*_eq_, which are the C-helix and the loop towards position 950. Additionally, the activation loop also sees a notable change with a ΔPB of 0.57. These results therefore show conformational changes between the two systems (see Figure 3E and Figure S6).

Hence, the phosphorylation of Tyr 1007 induces the flexibility of the region around position 950 and the mobility of the C-helix compared to the form without phosphorylation. The high flexibility of position 1007 is not really affected by its phosphorylation.

### 2.4. Impact of the Addition of Ruxolitinib on JH1 Apo-pTyr1007 Dynamics

**Figure 4:**
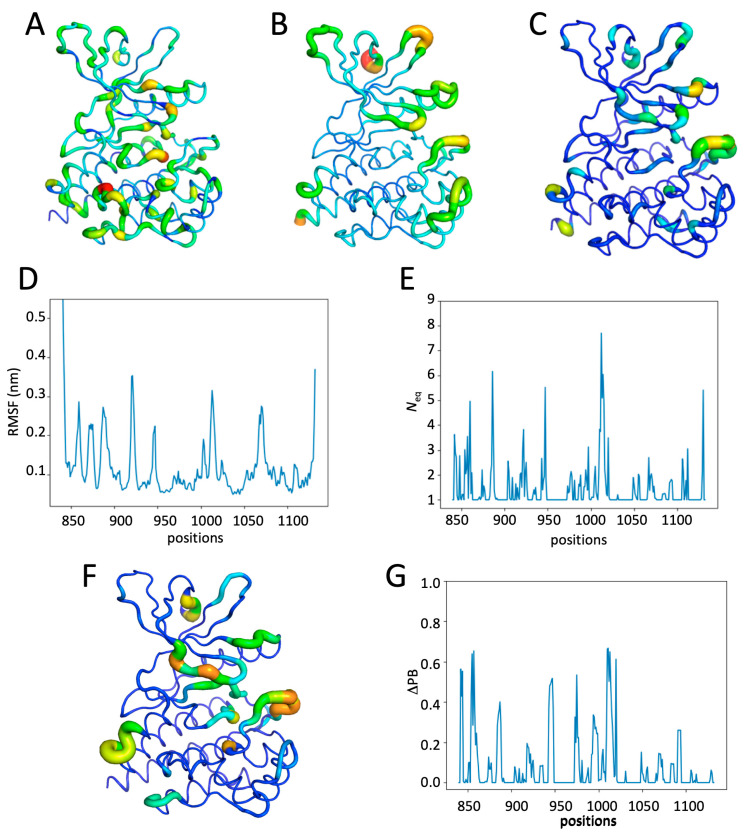
Analysis of the JH1 Ruxo-pTyr1007 system. (**A**) B-factor values, (**B**) RMSF values, and (**C**) *N*_eq_ values of the Ruxo-pTyr1007 system. (**D**) RMSF values and (**E**) *N*_eq_ values of Ruxo-pTyr1007 system along the residue position. ΔPB between the apo-pTyr1007 and Ruxo-pTyr1007 system (**F**) on the JH1 domain and (**G**) along the sequence (see the Figure 2 legend for more details).

The Ruxo-pTyr1007 system corresponds to the initial experimental structure (PDB ID 6VGL) [30]. It is therefore possible to compare the experimental values of the B-factors (see Figure 4A and Figure S7A) with the MD-derived metrics—the RMSF (see Figure 4B,D) and the *N*_eq_ (see Figure 4C,E). The analysis of B-factors gives a less clear image than the analyses of molecular dynamics simulations, with little difference between the different regions of the protein. Only the previously seen loop between ß-strands ß4 and ß5 is found with very high values, as in the catalytic loop (see Figure 4A). The distribution of RMSFs remains consistent with previous analyses (see Figure 4B,D). It first highlights the loop around position 950 between ß-strands ß4 and ß5, then the activation loop, the J-helix, followed by the hinge region, and the loop between ß-strands ß2 and ß3. The binding of the inhibitor did not affect the overall distribution of *N*_eq_ in the JH1 (see Figure 4C,E). There was no significant change in the position of rigid *N*_eq_ (proportion for *N*_eq_ equal to 1 is 52.9% and between 1 and 2 is 31.1%), leaving a similar proportion of positions with a *N*_eq_ greater than 2. On the other hand, the proportion of positions with a *N*_eq_ greater than 4 decreased by 2% (from 4% to 2%). The correlations observed between B-factors and RMSF, as well as B-factors and *N*_eq,_ are equivalent to the one classically observed in MD simulations between those three metrics, i.e., 0.5 for RMSF and 0.2 for *N*_eq_ (see Figure S7B,C) [42]. In fact, Ruxolitinib binding leads to interesting general changes in domain dynamics. Comparing the RMSF of apo-pTyr1007 and Ruxo-pTyr1007, the 890 region (C-helix) shows a small decrease (1 Å). Decreases from 0.2 to 0.5 Å are observed in almost all ß-strands and in smaller proportions everywhere (see Figure S8A). For *N*_eq_, between apo-pTyr1007 and Ruxo-pTyr1007, the maximum difference is still observed at the E-helix–D-helix loop (the *N*_eq_ value drops from 8.7 to 5.7; residue 950). While at this position, no significant difference in RMSF is noted (see Figure S8B). The activation loop also sees a decrease of 1.5 in its *N*_eq_ values at its N-terminal.

The ΔPB analysis between apo-pTyr1007 and Ruxo-pTyr1007 (see Figure 4F,G) shows the same regions as in the analysis between apo and apo-pTyr1007 (see Figure 3E,F), and it stays within the same range of values. The analysis of the PBs observed during the dynamics simulation (see Figure S9) shows that (i) the activation loop (see Figure 4E) presents a flexible region in both systems, and (ii) the loop between the D- and E-helices (see Figure S10) are stabilized. Indeed, in the presence of Ruxolitinib compared to the apo-pTyr1007 structure, an increase in rigid ß-sheet PB content (namely, PBs *f*, *b*, *d*, and *c*, see Figure S10A) is seen for the activation loop, and the stabilization of the α-helix (PB *m*, see Figure S10A) is seen for the E-helix–D-helix loop.

Interestingly, the binding of Ruxolitinib to apo-pTyr1007 (i.e., Ruxo-pTyr1007) seems to counterpoise the flexibility gain observed by the phosphorylation of Tyr10007. Indeed, the dynamics of JH1 are similar to those of inactive JH1 (apo system). The comparison of the distribution of RMSF and *N*_eq_ values between apo and Ruxo-pTyr1007 systems (see Figure S11A,B) is equivalent. Moreover, the analysis of the PBs of the E-helix–D-helix loop indicates that these two systems demonstrate the same behavior in this region (see Figure S11C). We can just note the stability of the G-rich loop and a very slight increase in the flexibility of the ß-sheet C-helix loop in Ruxo-pTyr1007 compared to apo JH1, but this is negligible.

Thus, Ruxolitinib seems to rigidify the JH1 domain by counteracting the effect of Tyr1007 mono-phosphorylation and stabilizes the flexible regions observed in apo-pTyr1007 simulations.

### 2.5. Impact of Multiple Phosphorylations and Ruxolitinib on JH1 Dynamics

**Figure 5:**
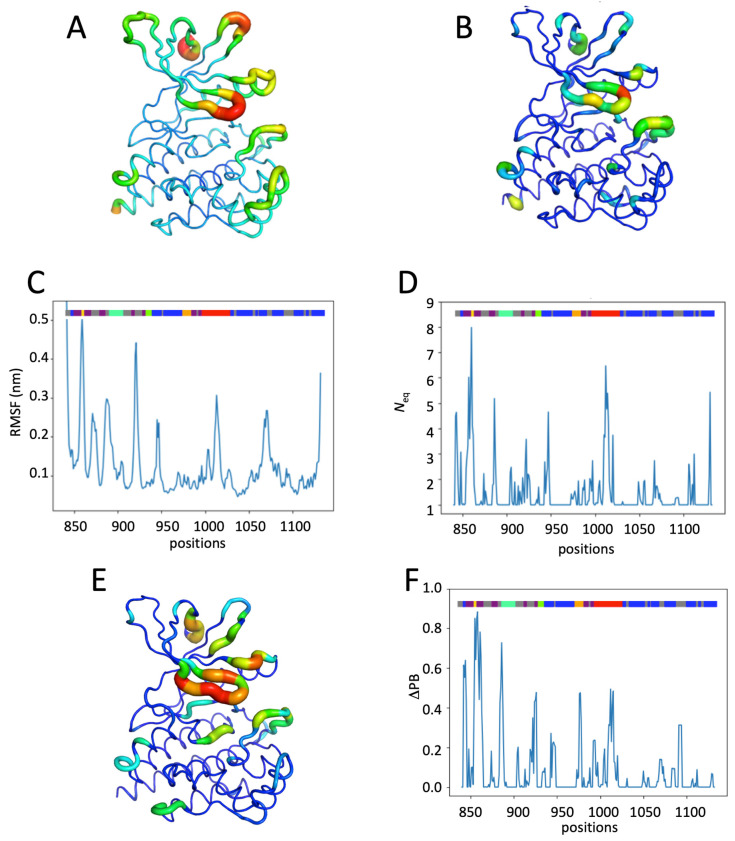
Analysis of the JH1 Ruxo-MultiPhosp system. (**A**) RMSF values and (**B**) *N*_eq_ values of the Ruxo-MultiPhosp system. (**C**) RMSF values and (**D**) *N*_eq_ values of the Ruxo-MultiPhosp system along the residue position. ΔPB between the Ruxo-pTyr1007 and Ruxo-MultiPhosp system (**E**) on the JH1 domain and (**F**) along the sequence (see the Figure 2 legend for more details).

Figure 5 shows the results of simulations with all phosphorylated sites and Ruxolitinib. RMSF values are particularly high at the G-rich loop (positions 858–860, increasing to 5.0 Å), the loop between ß-strands ß4 and ß5 (up to 4.5 Å), and to a lesser extent, at the activation loop (3.0 Å), the loop before helix-C, and the loop after Helix-J (see Figure 5A,C). The *N*_eq_ values show a disorder region for the G-rich loop (*N*_eq_ of 8), very high flexibility at the activation loop (*N*_eq_ of 6.5), and three other regions with a *N*_eq_ greater than 4 (outside the terminal areas). Thus, this system with five phosphorylated tyrosines shows a global distribution that is still close to the previous systems but with punctual position modifications. The ΔPB comparison shows that these high *N*_eqs_ values are associated with high differences in PB composition when comparing the Ruxo-pTyr1007 and Ruxo-MultiPhosp systems (see Figure 5E,F). Thus, the ΔPB of the G-rich loop is 0.9 (i.e., 45% differences in terms of PB composition), it is 0.7 (i.e., 35% differences) for the beginning of Helix-C, 0.6 for the first helix, and three other positions have a ΔPB of more than 0.4. The impact is therefore significant for two very close systems.

For JH1 activation to occur, several tyrosines must be phosphorylated. Therefore, the Ruxo-MultiPhosp system has been created in order to simulate a full system, but it is inhibited by Ruxolitinib. The analysis of the distribution of PBs therefore shows differences with previous systems (see Figure S12). The most striking example is the G-rich loop of Ruxo-MultiPhosp, which shows a greater global and local flexibility than in the Ruxo-pTyr1007 system (see Figure S13C). Therefore, the stabilization of the G-rich loop induced by Ruxolitinib is no longer present when the system is completely phosphorylated. From a distribution point of view in terms of PBs (see Figure S13C), there is not a drastic change in the PB types but a larger sample, sometimes causing a huge decrease in the main PB. For example, for Ruxo-pTyr1007 position 859 corresponds to 100% of PB *f*, but it is only 55% for Ruxo-MultiPhosp. Similarly, at position 862, we go from 98% of PB *c* to 54%. The other positions of the motif thus often see the majority of PB become the second or third principal.

Hence, polyphosphorylation simulates a flexible fully active system. The stabilization of the G-rich loop is no longer present when the system is fully phosphorylated.

## 3. Discussion

**Figure 6:**
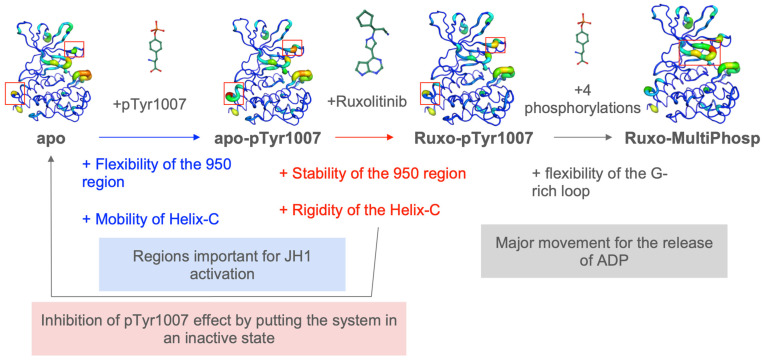
Summary diagram of Ruxolitinib action on the JH1 domain.

Figure 6 summarizes all the information obtained during this analysis (see also Figure S14 for an analysis of different clusters observed between systems). In the inactive, ligand-free state (apo), JH1 exhibits the high local flexibility of the activation loop (see Figure 2 and Figure S3). The mono-phosphorylation of residue Tyr 1007 (apo-pTyr1007) induces a slight stabilization of the loop, but it also induces a significant increase in the RMSF and *N*_eq_ of the C-helix, as well as the *N*_eq_ of the E-helix–D-helix loop around residue 950 (see Figure 3 and Figure S6). These differences in flexibility, that we can hypothesize are caused by the phosphorylation of Tyr 1007, could have an important impact on JAK2-JH1 activation, induced by the important role that the E-helix plays in the formation of the ATP binding site [30,34]. The helix movements caused by the flexibility of the loops could favor protein–protein or protein–substrate interactions.

When Ruxolitinib binds to the mono-phosphorylated system (Ruxo-pTyr1007), it induces the rigidification of the E-helix–D-helix and C-helix loops (see Figure 4 and Figures S8, S10 and S11). This stabilization is also shown by the greater diversification of conformation of apo-pTyr1007, when the Ruxolitinib is not present in the binding site (see the clusters in Figure S14). The presence of Ruxolitinib allows the dynamic behavior to be similar to that of the JH1 domain alone, which is supposedly inactive (see Figure S11). However, the G-rich loop appears to be more stable in the presence of Ruxolitinib (Ruxo-pTyr1007). This difference could be due to the stabilization of the loop by interactions with Ruxolitinib, shown by the “closed” conformation of the loop that is never reached in the presence of the ligand (see Figure S14). Altogether, this suggests a mechanism that can lead to the inhibition of JAK2 activity with Ruxolitinib. Its binding not only prevents ATP binding but also stiffens the entire JH1, which is unfavorable for JAK2 activation.

When the system is fully phosphorylated (Ruxo-MultiPhosp system; see Figure 5), the G-rich loop, which has the role of stabilizing interactions with ATP [29,33], becomes very flexible. We can hypothesize that the total phosphorylation of JH1 could lead to the release of ATP in a normal activation by the movement of this loop. On the contrary, we also observe that the C-helix does not change its conformation between the Ruxo-MultiPhosp and the Ruxo-pTyr1007 systems (see Figure S14). A shift in the C-helix is also observed in the comparison of the crystallized structure of the active and inhibited form of JAK2-JH1 [38]. This strengthens the hypothesis that Ruxolitinib inhibits JH1 by returning the system to the inactive state.

To conclude, we propose here a dynamic study of the JH1 domain of the JAK2 protein and propose first insights that could explain, at least partially, the inhibition observed by the binding of Ruxolitinib. However, more information on the dynamics of JH1 in ATP binding is needed to draw conclusions on the behavior of the active state of JH1. Interesting potential directions for this work would be to optimize the effect of Ruxolitinib by modifying some of its groups, thus evaluating their contributions, and to extend their size by Fragment-Based Drug Design. Similarly, it would be particularly interesting to observe the effect of other ligands, such as Pacritinib (whose complex is available, PDB id 8BPV [33]). In addition, including the JH2 domain in further studies could be very helpful in fully understanding the long-range effect of this mutation onto the kinase domain and how the inhibitor can counterbalance this effect.

## 4. Materials and Methods

### 4.1. Selection of the JH1 Domain and the Different Systems

Janus kinase 2 structures were downloaded from the Protein Data Bank website (https://www.rcsb.org, (accessed on 29 June 2024)) [43]. The JH1 domain structure was extracted from PDB id 6VGL [30]. The structure was analyzed using classical approaches such as MolProbity [44] and visualized with PyMOL v.2.4.0 software (https://pymol.org/2/, (accessed on 15 June 2024)) [45,46,47].

The JH1 domain from 6VGL has one phosphorylation (at position 1007) and is in complex with Ruxolitinib. Four JH1 systems were prepared starting with this initial structure, as follows: (i) the wild-type system, named “apo”, not phosphorylated and without Ruxolitinib; (ii) “apo-pTyr1007”, having the resolved phosphorylated tyrosine at position 1007 but without Ruxolitinib; (iii) “Ruxo-pTyr1007”, which contains the same phosphorylation (at position 1007 and is in complex with Ruxolitinib); and (iv) “Ruxo-MultiPhosp”, the system with all defined phosphorylation positions, as proposed in UniProt human JAK2 entry O60674 (https://www.uniprot.org/uniprotkb/O60674/entry (accessed on 29 May 2024)), while being in complex with Ruxolitinib. All five phosphorylations were performed using CHARMM-GUI [48] (https://www.charmm-gui.org/, (accessed on 10 May 2024)).

### 4.2. Molecular Dynamics

Molecular Dynamics (MD) simulations were performed using GROMACS 2023.4 software [49] with the CHARMM-36 force field [50] for all the systems, with similar parameters and the same protocol. The inhibitor parameters were generated with the help of the CGENFF tool [51]. Before starting any simulation experiments, each system was energy-minimized for 50,000 steps of the steepest descent until the maximal force reached 1000 kJ/mol/nm, using the Gromacs suite. Each system was soaked in a cubic simulation box with TIP3P water molecules. Ions were also added in order to neutralize the system. The MD protocol had been standardized through our previous works [41,52]. After 100 picosecond (psec) of each equilibration (with position restraints on the protein), each system was simulated through multiple classical independent production runs with 4 replicates of 250 nanoseconds, as performed in [41]. The equilibration consists of one step with a NVT system and one step with a NPT system. During these two steps, the protein is totally constrained and unable to move, while the equilibration affects the water molecules. Constraints are gradually removed to progressively equilibrate the system. Molecular conformations were saved every 10 picoseconds for downstream analysis. A total of 1µs of the MD simulation was produced for each system.

Trajectory analyses were performed with the GROMACS software 2023.4, in-house Python 3 and R scripts. The root mean square deviations (RMSD) and root mean square fluctuations (RMSF) were calculated on Cα atoms alone.

### 4.3. Molecular Dynamics Analysis

The analysis of MDs is performed using classic approaches, such as the RMSD and RMSF, and other more innovative ones such as PBxplore [53].

The RMSD (Root Mean Square Deviation) quantifies the structural variations in the dynamics by comparing each frame to a reference structure, i.e., the starting frame. For each frame, an average of the differences between the reference positions and the positions of the current frame is performed in order to have an RMSD value per unit of time.

The RMSF (Root Mean Square Fluctuation) is similar to the RMSD, by determining the fluctuation of each residue following the same principle as for the RMSD, i.e., a comparison with a reference. But this time, it is the average position of each residue, and thus, the measure of the difference between the current position and the average position in order to have a flexibility value per position.

The assignment of secondary structures was performed using the Dictionary of Secondary Structure of Protein (DSSP) [54,55]. DSSP provides eight states of description (α-helix, π-helix, 3_10_-helix, β-strands, β-turns, bents, β-bridge, and coil). Thus, from the trajectory file generated by GROMACS, DSSP assigns the secondary structure element for each time step. This analysis allows us to easily see the stability of the protein secondary structure elements as a function of time.

Protein Blocks (PBs) are a structural alphabet composed of 16 local prototypes [39,56,57]. Each specific PB is characterized by the φ, ψ dihedral angles of five consecutive residues with each PB assignment focused on the central residue. Obtained through an unsupervised training approach and performed on a representative non-redundant databank, PBs give a reasonable approximation of all local protein 3D structures [58]. PBs are very efficient in tasks such as protein superimpositions [59,60] and MD analyses [61,62]. They are labeled from *a* to *p* as follows: PBs *m* and *d* can be roughly described as prototypes for the α-helix and central β-strand, respectively. PBs *a* to *c* primarily represent β-strand N-caps, with PBs *e* and *f* representing β-strand C-caps. PBs *a* to *j* are specific to coils; PBs *k* and *l* are specific to α-helix N-caps; and PBs *n* to *p* are specific to α-helix C-caps. PB assignment was carried out using our PBxplore tool (https://github.com/pierrepo/PBxplore, (accessed on 29 November 2024)) [53].

PB assignments are performed for each residue of the JH1 domain and over every snapshot extracted from the MD simulations. The equivalent number of PBs (*N*_eq_) is a statistical measurement, similar to entropy, that represents the average number of PBs for a residue at a given position. *N*_eq_ is calculated as follows [39]:

$$N_{eq}=exp\left(-\sum_{x=1}^{16}f_{x}\ln f_{x}\right)$$

where *f_x_* is the probability of PB *x*. A *N_eq_* value of 1 indicates that only one type of PB is observed, while a value of 16 is equivalent to a random distribution. To underline the main differences between one system and another for each position, the absolute difference Δ*N_eq_* between corresponding *N*_eqs_ values was computed.

However, since the same Δ*N*_eq_ value can be obtained with different types of blocks in similar proportions, we have defined a complementary measure, ΔPB, that evaluates a change in PB profile, by calculating the absolute sum of the differences for each PB between the probabilities of PB *x* to be present in the first and second forms (*x* goes from PB *a* to PB *p*). ΔPB is calculated as follows [41]:

$$\Delta PB=\sum_{x=1}^{16}\left|{(f}_{x}^{1}-f_{x}^{2})\right|$$

where *f^1^_x_* and *f^2^_x_* are the percentages of occurrence of a PB *x* in, respectively, the first and the second system. A value of 0 indicates a perfect PB identity across the first and second systems, while a score of 2 indicates the maximum total difference.

PBxplore also uses WebLogo to provide a dedicated PB logo output [63].

## Data Availability

Molecular dynamics trajectories are available upon request.

## Supplementary Materials

The following supporting information can be downloaded at: https://www.mdpi.com/article/10.3390/ijms26083727/s1.
