# Supplementary material for: Impact of Ruxolitinib Interactions on JAK2 JH1 Domain Dynamics
Source: Int J Mol Sci. 2025 Apr 15;26(8):3727. doi: 10.3390/ijms26083727 (PMC12028094; doi:10.3390/ijms26083727)
Supplement: Supplementary file 1 [file ijms-26-03727-s001.zip › ijms-3545007-supplementary.pdf]

Title: Impact of Ruxolitinib interactions on JAK2 JH1 domain dynamics.

Authors: Hong Nhung Vu, Ragousandirane Radjasandirane, Julien Diharce and Alexandre G. de Brevern

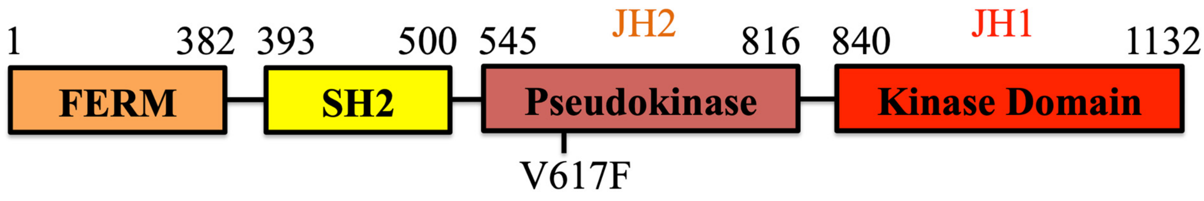

Figure S1. *Positions of the four consecutive domains of JAK2.*

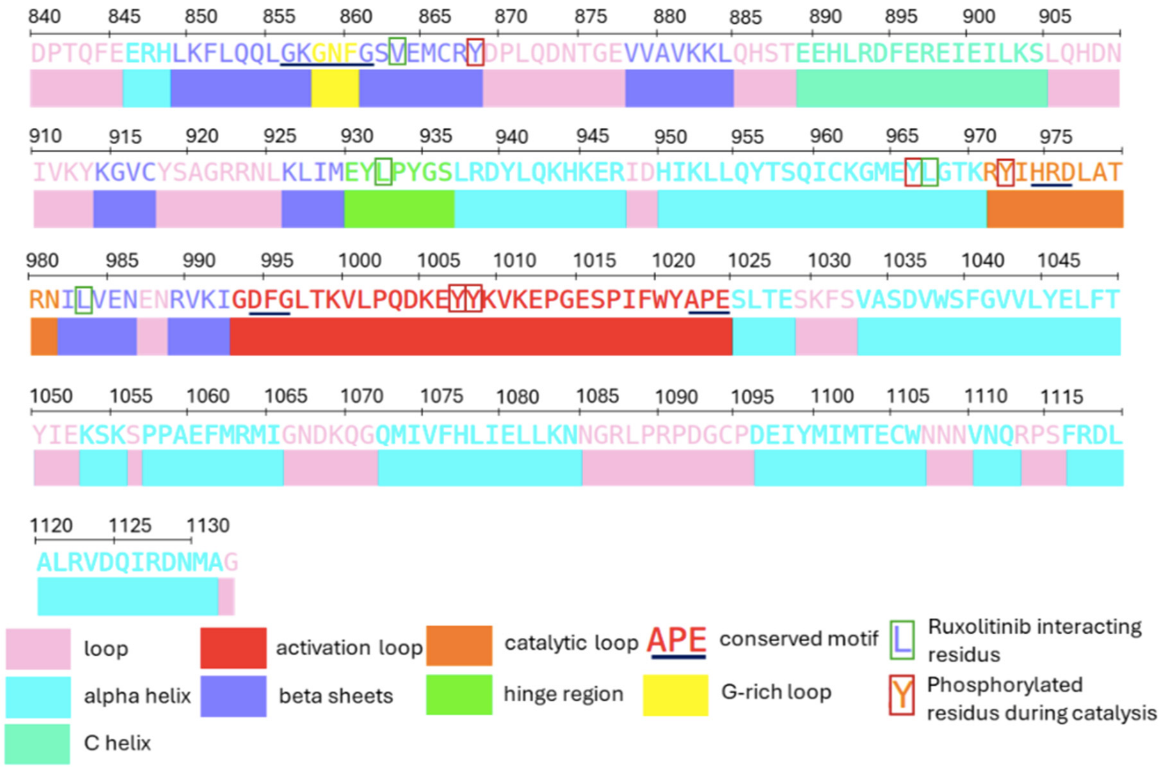

Figure S2. *The different parts of the JH1 domain.*

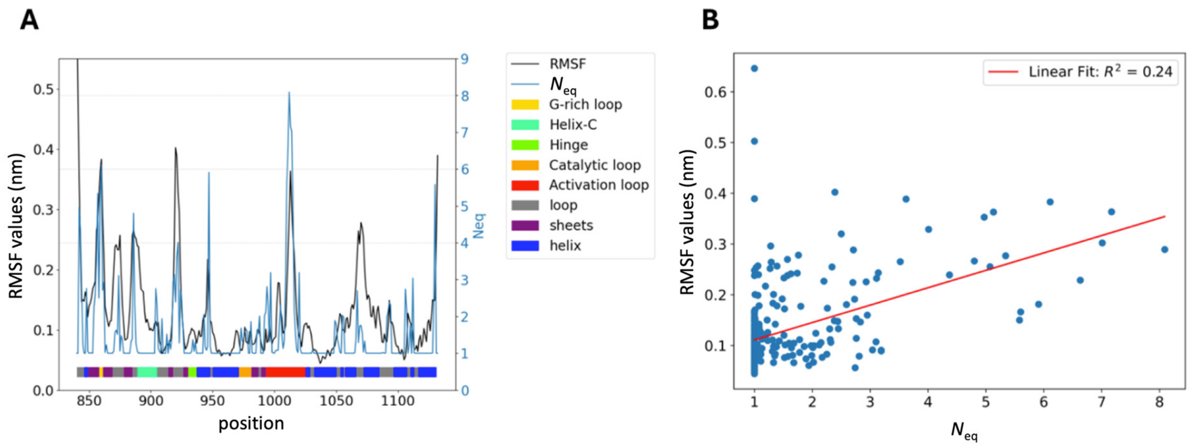

Figure S3. *Comparison of RMSF and N_{eq} values for the JH1 apo*. (A) RMSF (blue line) and N_{eq} values (black line) plotted along the sequences. (B) Correlation of the two distributions.

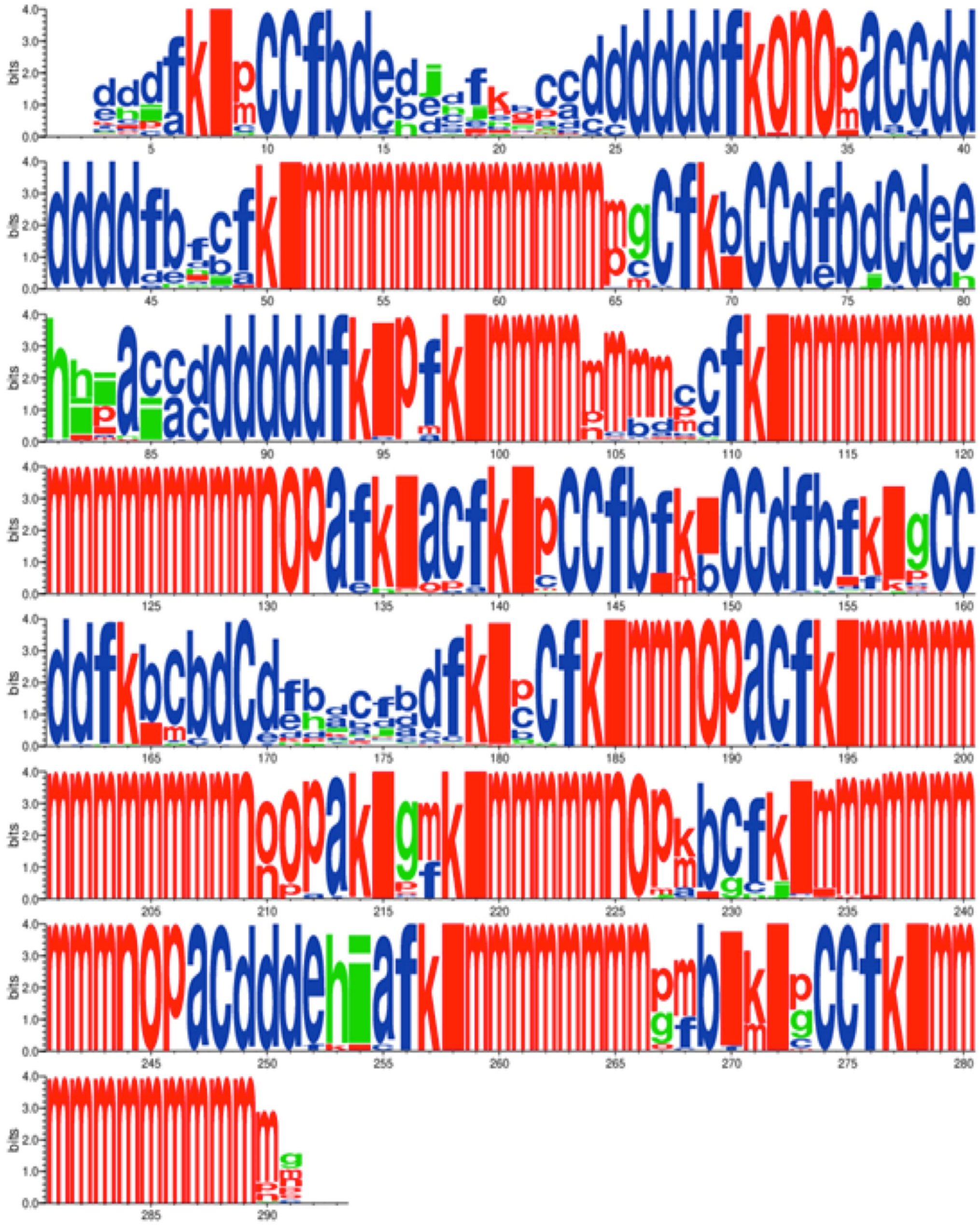

Figure S4. *JH1 apo domain Molecular Dynamic simulations translated into Protein Blocks and shown with WebLogo.*

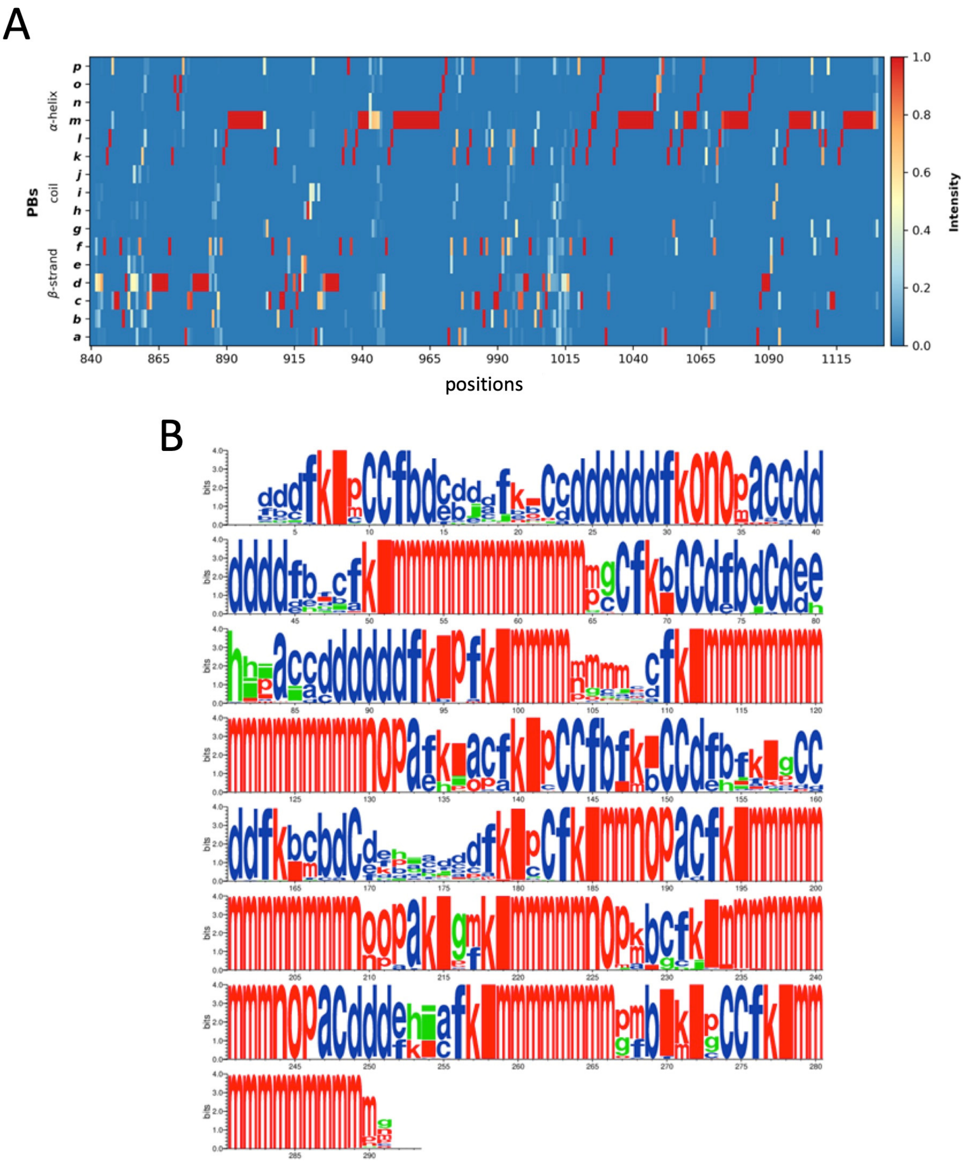

Figure S5. *JH1 apo-pTyr1007 domain Molecular Dynamic simulations (A) translated into Protein Blocks and (B) shown with WebLogo.*

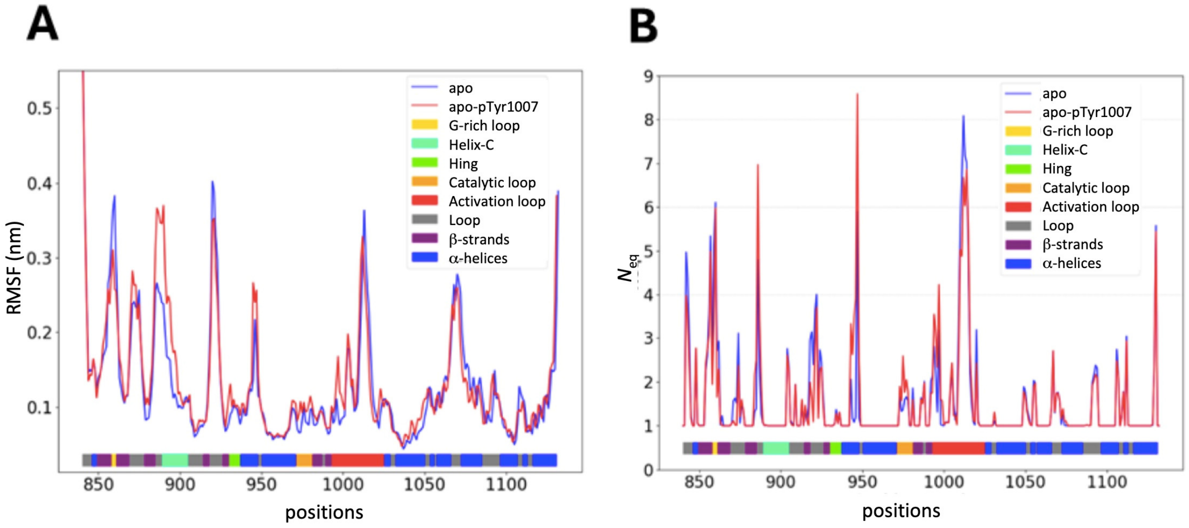

Figure S6. *Comparison between apo and apo-pTyr1007 system.* (A) Superimposition of RMSF values and (B) of N_{eq} values. JH1 apo is the blue line; JH1 apo-pTyr1007 is the red line.

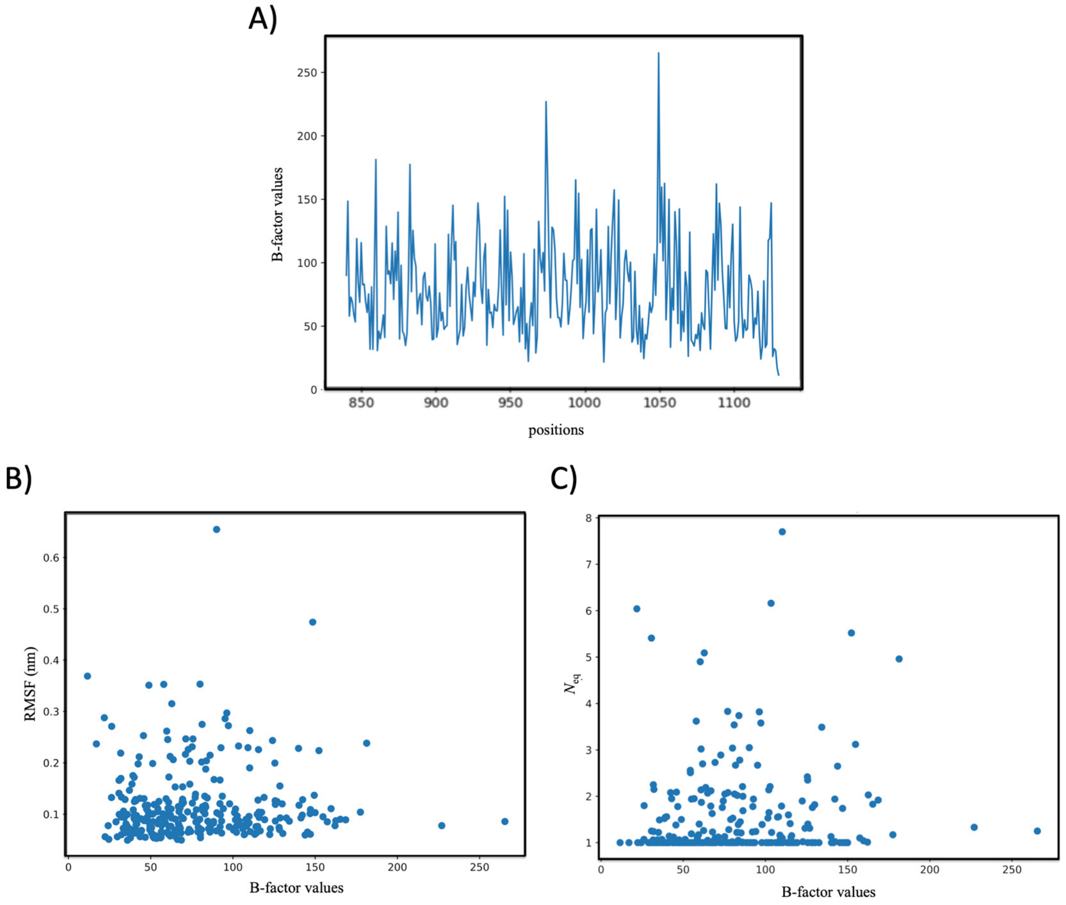

Figure S7. *Analysis of B-factor values*. (A) Raw values of B-factor. (B) B-factor values *vs.* RMSF values. (C) B-factor values *vs.* RMSF values.

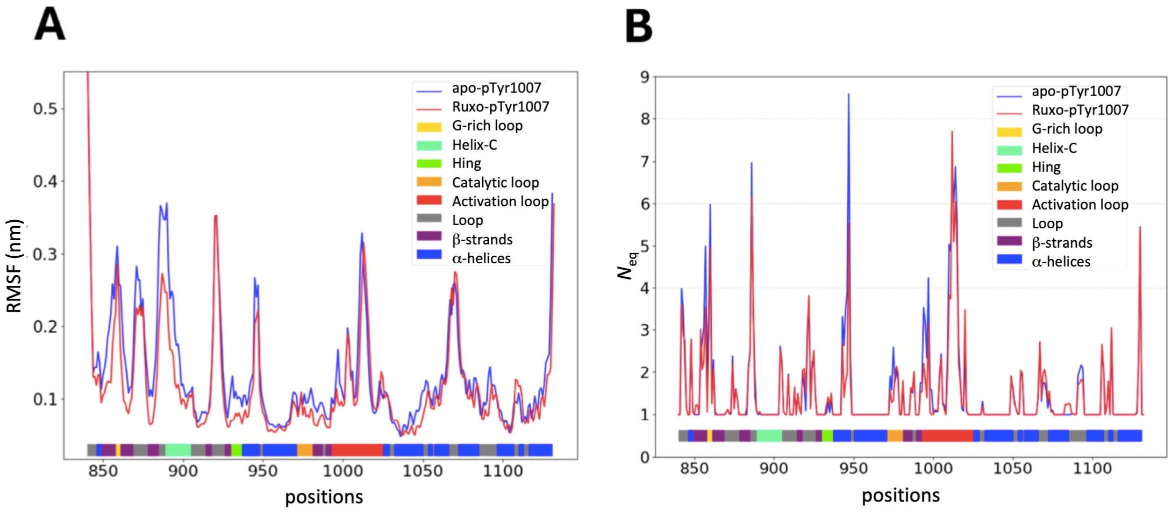

Figure S8. *Comparison between apo-pTyr1007 and Ruxo-pTyr1007 system*. (A) Superimposition of RMSF values and (B) of N_{eq} values. JH1 apo-pTyr1007 is the blue line; JH1 Ruxo-pTyr1007 is the red line.

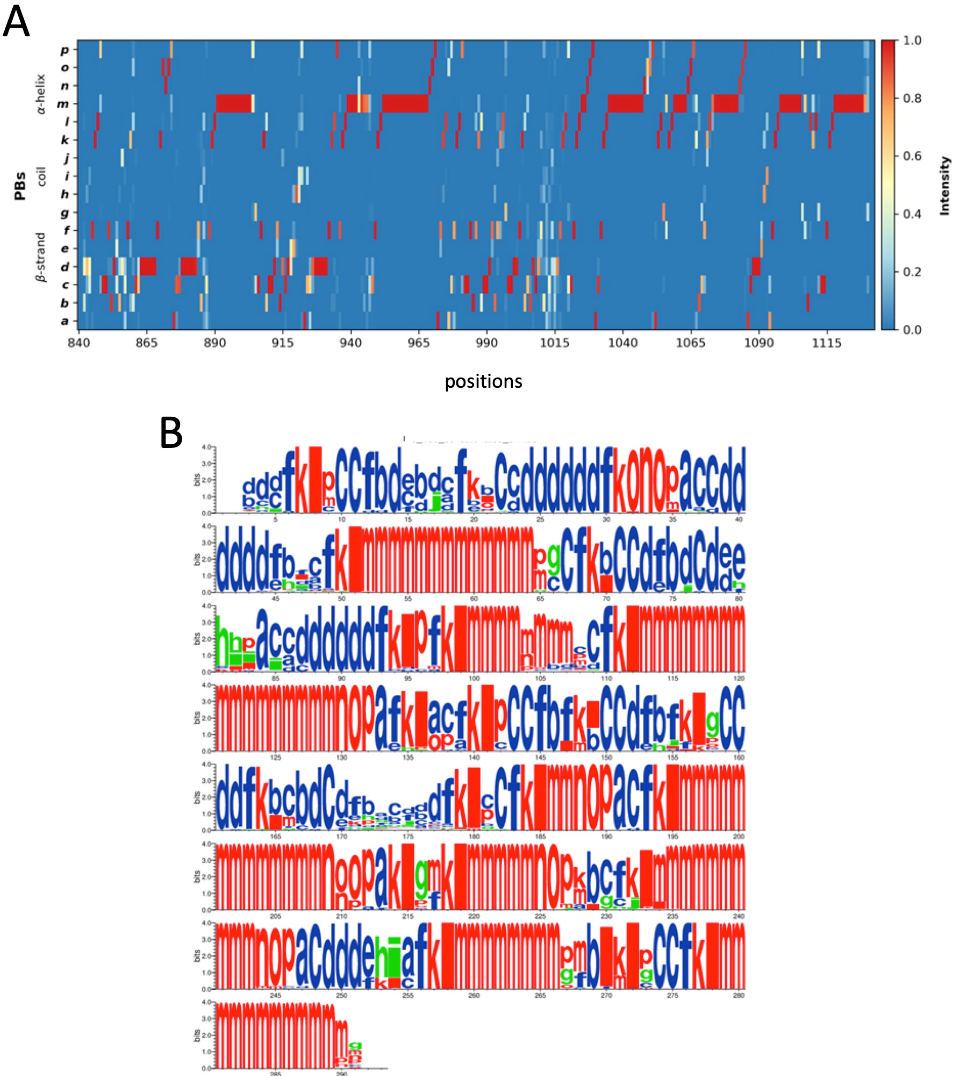

Figure S9. *JH1 Ruxo-pTyr1007 domain Molecular Dynamic simulations (A) translated into Protein Blocks and (B) shown with WebLogo.*

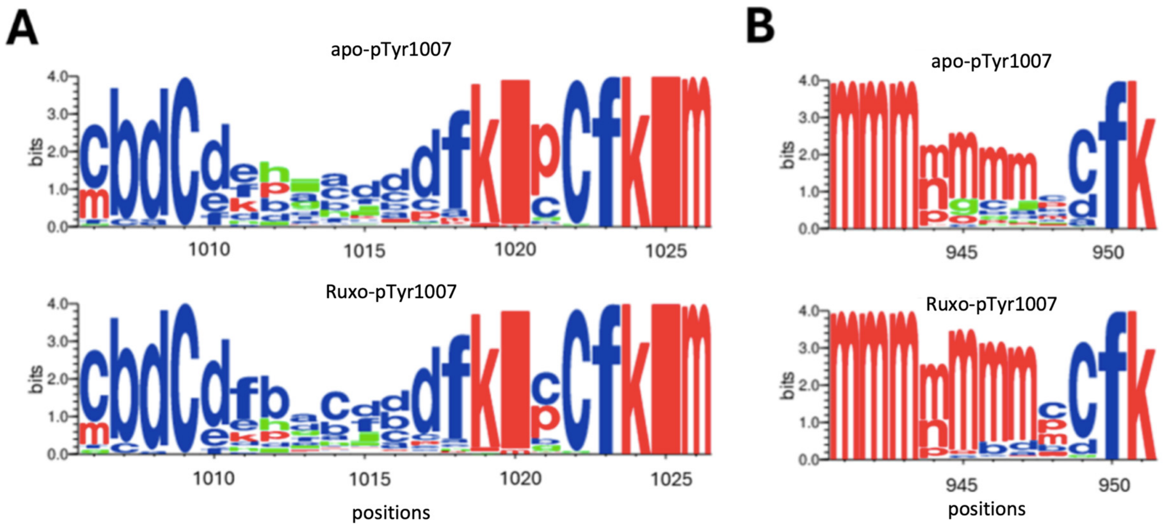

Figure S10. *Comparison of apo-pTyr1007 (top) and Ruxo-pTyr1007 (bottom) system.* (A) The activation loop (positions 1005 to 1025) and (B) the region corresponding to helices-E and D (positions 940 to 950).

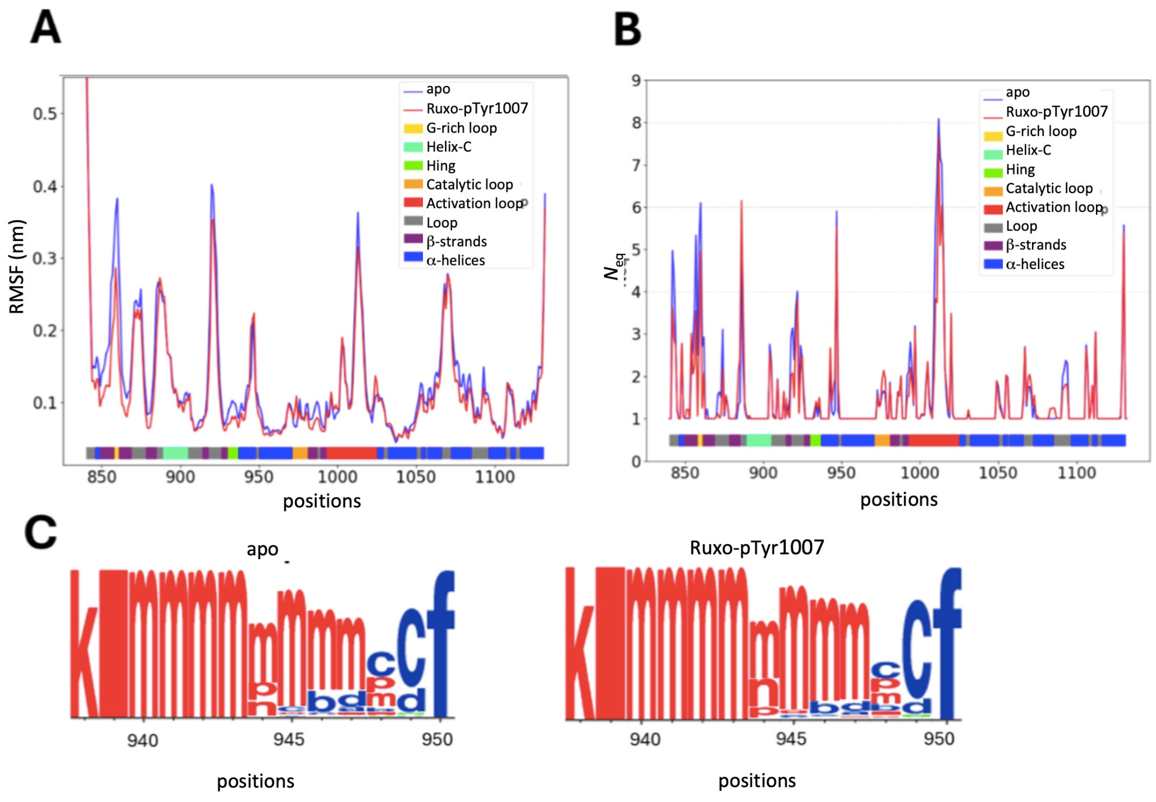

Figure S11. *Comparison between apo and Ruxo-pTyr1007 system.* (A) Superimposition of RMSF values and (B) of N_{eq} values. JH1 apo is the blue line; Ruxo-pTyr1007 is the red line. (C) PB distribution for the loop between D-helix and E-helix (positions 940 to 950).

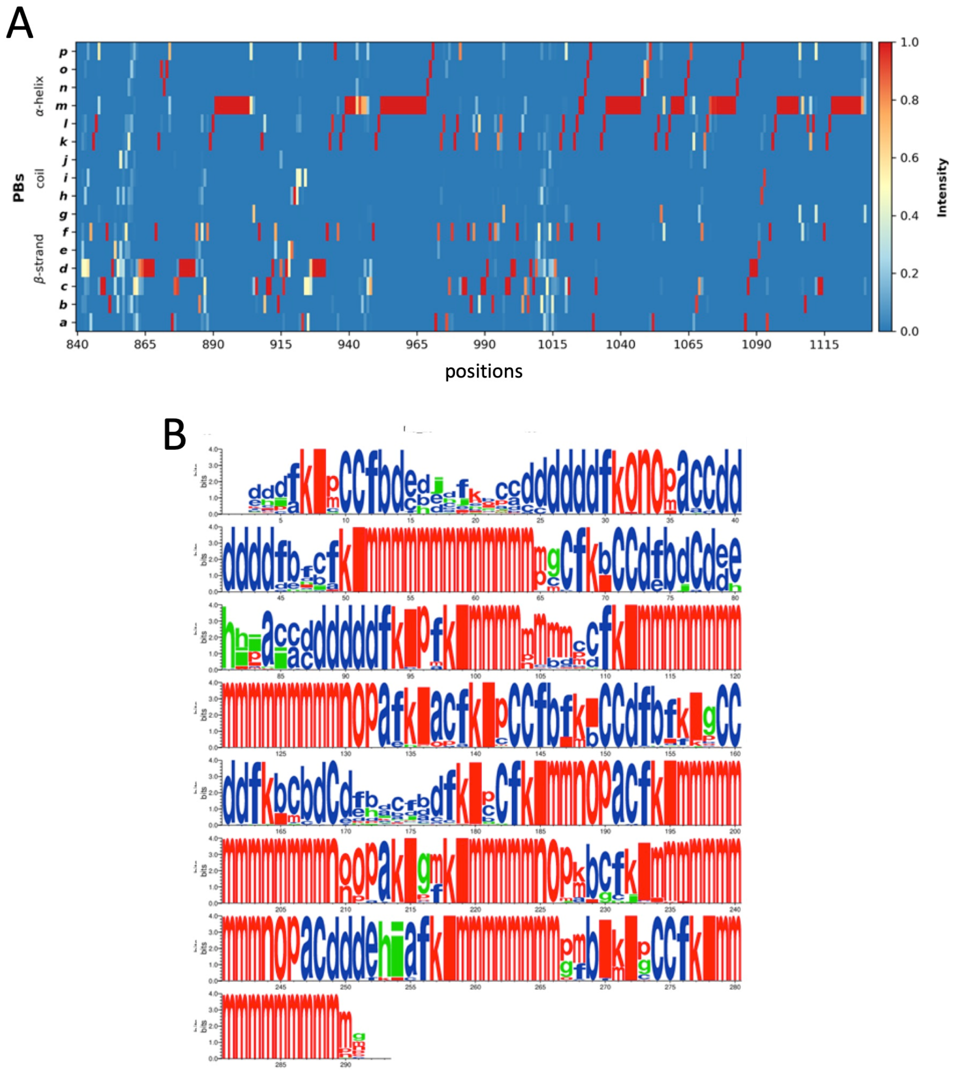

Figure S12. *JH1 Ruxo-MultiPhosp domain Molecular Dynamic simulations (A) translated into Protein Blocks and (B) shown with WebLogo.*

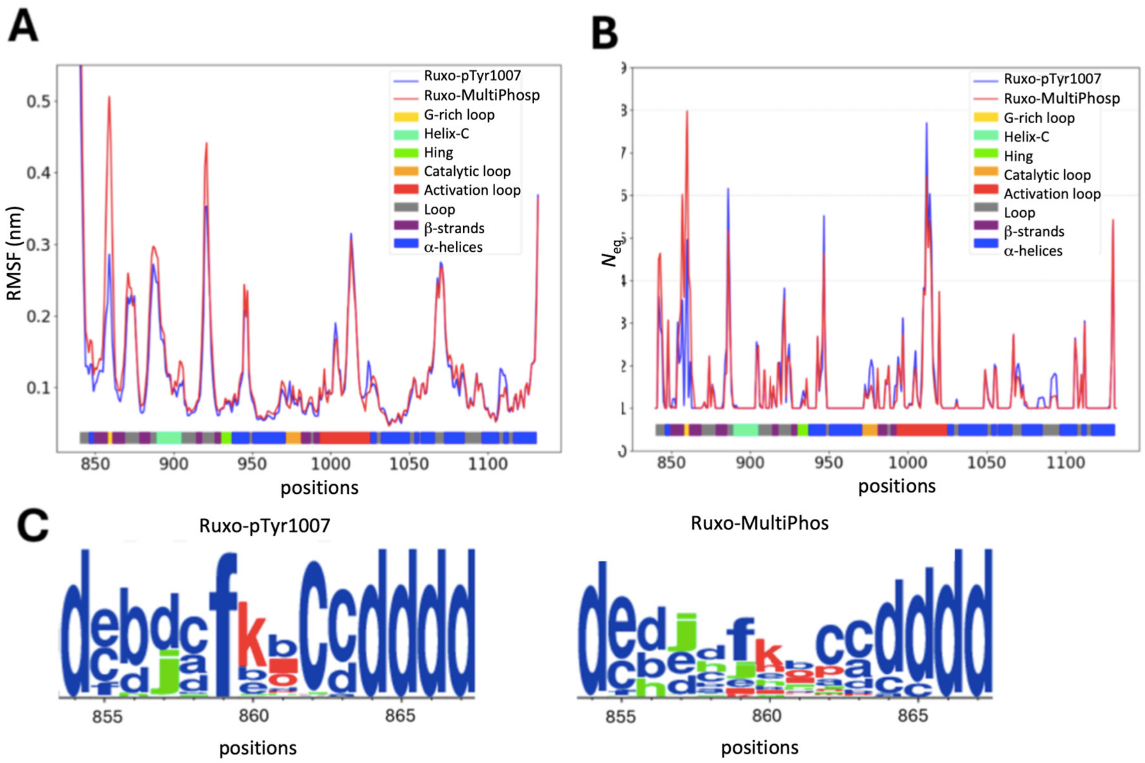

Figure S13. *Comparison between Ruxo-pTyr1007 and Ruxo-MultiPhosp system.* (A) Superimposition of RMSF values and (B) of N_{eq} values. JH1 Ruxo-pTyr1007 is the blue line; Ruxo-MultiPhosp is the red line. (C) PB distribution for the G-rich loop (positions 855 to 865).

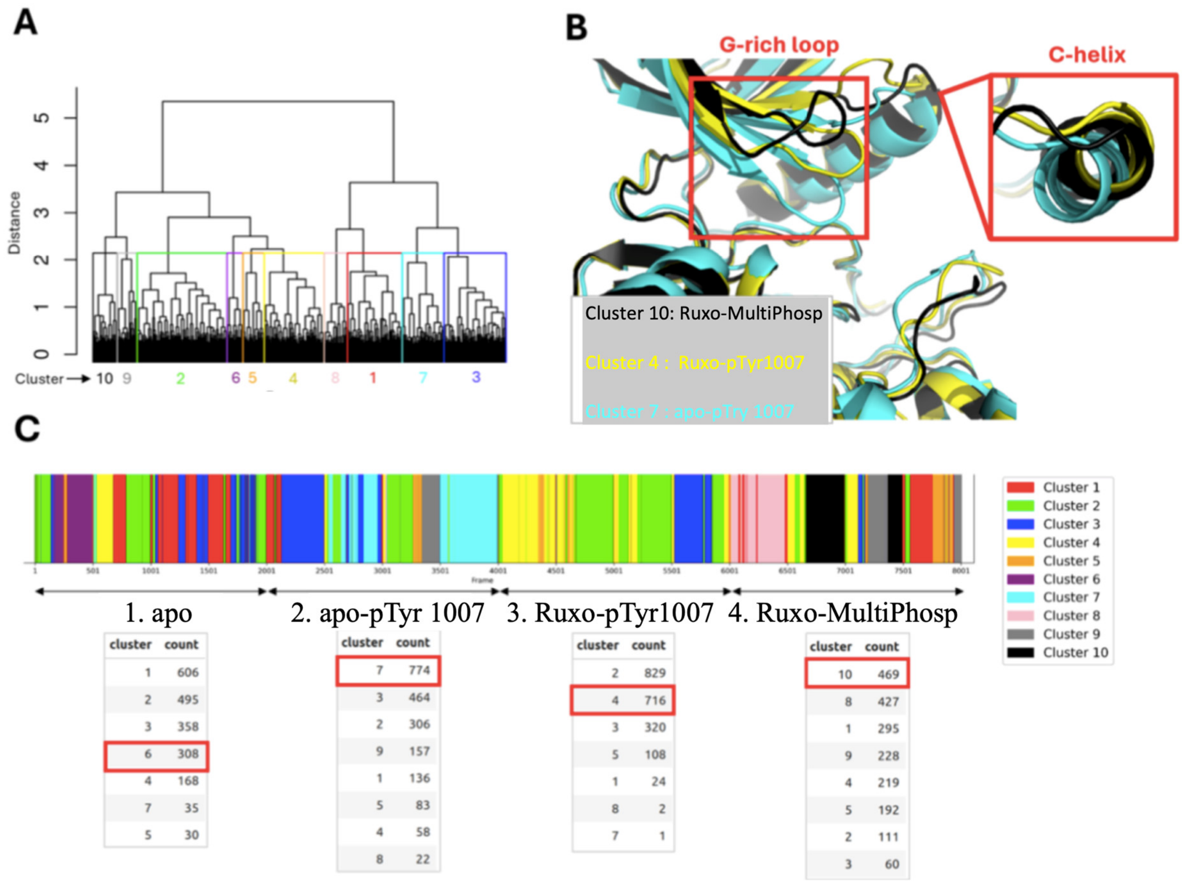

Figure S14. *Cluster analysis.* (A) Dendrogram (hierarchical clustering with Ward metric) of all the simulations. (B) Superposition of representative clusters of different systems. (C) Cluster distribution along the different systems. The table gives the occurrence observed for every cluster per system. The most specific cluster of each system is framed in red.

For the clustering analysis, all the trajectories of 4 systems were concatenated with GROMACS to have only one simulation of 4 µs. The snapshots were compared on the basis of the RMSD performed using the GROMACS *mdtraj* command. The hierarchical classification was performed using the R software and its *hclust* function with the Ward method (Ward.D2) that focuses on minimizing the total variance (the sum of the squares of the deviations) within the cluster. 10 clusters were generated (see Figure A). The obtained clusters are shown on Figure A.C: cluster 7 for the apo-pTyr1007 system, cluster 4 for the Ruxo-pTyr1007 system and cluster 10 for the Ruxo-MultiPhosp system (see Figure A.B). Cluster 1 is the most dominant in the apo system but it is also present in significant quantities in the other clusters.

The clusters in the Ruxo-pTyr1007 system are less diverse than in the apo-pTyr1007 system, which confirms the stabilization of JH1 by the fixation of Ruxolitinib.

We evaluated the difference between the two systems by comparing the representative cluster of each system, which is an abundant cluster in this system and very little present in the other. The comparison of cluster 4, representative of the apo-pTyr1007 system, and cluster 7, representative of the Ruxo-pTyr1007 system showed that they diverged at 3 regions including: the C-helix, the Glycine-rich loop, and the activation loop. We observe that in the presence of Ruxolitinib, the rapprochement of the C-helix towards the activation loop and the closure of the Glycine-rich loop are prevented.

We also note a similar position of the C-helix between Ruxo-pTyr1007 and Ruxo-MultiPhosp. However, the Glycine-rich loop is more open in Ruxo-pTyr1007 that is consistent with a better flexibility of this region that we had observed in the previous sections.
